# Contemporary Insights Into HIV Esophagitis: Pathogenesis, Therapeutic Strategies, and Prognostic Outcomes

**DOI:** 10.7759/cureus.60788

**Published:** 2024-05-21

**Authors:** Dhruvikumari D Sharma, Peter Girgis, Dhruv Gandhi, Sparshitha Adapa, FNU Karishma, Gurvir Kaur, Gohul P Balasingh, Mostafa Mohamed Ismail Elnimer

**Affiliations:** 1 Medicine, Avalon University School of Medicine, Willemstad, CUW; 2 Internal Medicine, Ross University School of Medicine, Bridgetown, BRB; 3 Internal Medicine, K. J. Somaiya Medical College, Mumbai, IND; 4 Internal Medicine, Osmania Medical College, Hyderabad, IND; 5 Internal Medicine, Ghulam Muhammad Mahar Medical College, Khairpur, PAK; 6 Internal Medicine, American University of Antigua, Los Angeles, USA; 7 Internal Medicine, Tbilisi State Medical University, Tbilisi, GEO; 8 Medicine, Kafr Elsheikh Liver Research Center, Kafr El-Shaikh, EGY

**Keywords:** prognostic outcomes, therapeutic strategies, aids, esophageal candidiasis, clinical management, prognosis, treatment options, pathogenesis, esophagitis, hiv

## Abstract

Opportunistic infections caused by various bacteria, viruses, fungi, or parasites can cause esophagitis. The fungus *Candida albicans* is often believed to be the thief behind this disorder. This condition's distinctive signs include the process of inflammation and the development of esophageal ulcers. The underlying immunodeficiency condition in HIV/AIDS patients, especially those in the late stages of the disease, may lead to severe illness or even death if the lowered immune system can no longer combat common infections. These individuals are, therefore, more at risk of contracting diseases like Candidiasis since they already have weakened immune systems. Furthermore, bacteria and mycobacteria can cause esophagitis in the same way that viruses can. Tobacco use, alcohol drinking, and nutritional deficiency are three additional problems that can lead to an HIV esophagitis infection. Complaints of inability to swallow, suffocating feeling or discomfort behind the breastbone, and painful swallowing are the primary symptoms of the patients. White plaques or ulcers observed in the esophagus during an endoscopy can be biopsied for further examination. The presence of *C. albicans* hyphae and inflammatory infiltrates in these samples confirms the diagnosis of HIV-associated esophagitis. Treatment involves the use of antifungal medications and addressing any underlying causes of esophagitis, which is linked to AIDS. For superficial to moderate infections, fluconazole is typically used first. If the disease is severe or recurs after treatment, intravenous amphotericin B may be necessary. Patients with recurring oral symptoms of HIV esophagitis might also need to take antifungal drugs as a preventative measure.

## Introduction and background

The non-enveloped double-stranded RNA molecule is the defining feature of HIV, a retrovirus. Recognized as an epidemic on a global scale, HIV infection has affected communities around the world by weakening immune systems and triggering a cascade of linked illnesses [1]. Most scientists agree that the virus first infected non-human primates in Central Africa before affecting people [2]. HIV infection may spread during pregnancy, via blood-to-blood contact (for example, sharing needles), and, most often, through sexual engagement [3]. Headaches, enlarged lymph nodes, ulcers on mucous membranes, excessive fatigue, low energy, elevated body temperature, and inflammation of the throat are typical symptoms of HIV infection [4]. Because it explicitly targets CD4 T cells, the immune system's natural defense mechanism, the virus can generate chronic persistent infections by weakening the body's ability to overcome the infection. The immune system becomes highly activated when an individual contracts HIV, leading to consistently high levels of cytokines that promote inflammation [5]. Several long-term complications of HIV infection, including heart disease, kidney disease (also called HIV-associated nephropathy), liver disease, neurological disorders (including dementia and neurocognitive disorders), gastrointestinal disorders, and cancer, are caused by cytokines. Reduced levels of pro-inflammatory cytokines after starting highly active antiretroviral treatment lessen the probability of inflammatory complications. Even after they have had enough therapy, HIV-positive people are still at risk for certain diseases [5,6].

According to the study, upper gastrointestinal symptoms are common in HIV-infected individuals, even when they strictly adhere to highly active antiretroviral therapy (HAART) [7]. Many factors may be at play here, but medication resistance and decreased drug absorption are two potential explanations. The inflammatory and infectious condition known as HIV esophagitis is one example of this type of illness [8]. Opportunistic infections, including candidiasis and other fungal diseases, as well as viruses like cytomegalovirus, and herpes simplex virus, are the most common causes of HIV esophagitis. The hallmark signs include dysphagia, nausea, acid regurgitation, heartburn, early fullness, and odynophagia. It is essential to gather a thorough record of disease-specific gastrointestinal symptoms to give adequate and timely medication, which reduces adverse outcomes and improves the overall prognosis [9].

The gold standard for identifying HIV esophagitis in people exhibiting esophageal symptoms is endoscopy. White, plaque-like lesions on the mucosa can be seen by endoscopy, making it a powerful diagnostic tool. Endoscopy is also accurate in diagnosing esophageal or gastric-related symptoms [10]. Additionally, it can take a biopsy, which, for the majority of AIDS patients, is the most appropriate course of action for further molecular analysis using histology, cultures, or specific strains to identify certain particular symptoms' origin. An appraisal of esophagitis in the context of the HIV case is provided in this article. Speaking about the etiology, mechanisms, therapy, and prognosis of HIV esophagitis, the study offers essential and particular knowledge [11].

## Review

Epidemiology and pathogenesis

The side effect of antiretroviral therapy is esophagitis, but its frequency and severity are exceptionally high among the AIDS population. *Candida albicans* infections are the most common cause of esophagitis. Still, it can also result from a virus, which is categorized as herpes or cytomegalovirus, or a disease not caused by the host itself. Numerous observational studies have proved the relationship between bacterial infections such as *Mycobacterium avium* and esophagitis. The grim prognosis is a routine for esophageal symptom sufferers whose average survival time from the diagnosis is five months with all that data ranging from one to 13 months. Candida infection and colonization are possible outcomes of a cell-mediated immune deficit in the esophageal epithelial layer. The development of white-yellow plaques is caused by the proliferation of Candida and its attachment to the mucous membrane of the esophagus. These plaques can be observed across the entire esophagus. Groups of squamous cells that differentiate from the primary mucosal tissue bordered by squamous cells characterize desquamated parakeratosis. However, this finding applies to more than only esophageal candidiasis [12].

According to a study, herpes simplex virus esophagitis may occur either when the virus reactivates and travels straight to the vagus nerve or when an infection moves from the mouth to the esophagus. In several cases, it has been associated with oropharyngeal ulcers and herpes labialis. The mucosa of the lower section of the esophagus is affected by ulcers, which are well-defined (less than 2 cm) and have a unique “volcano-like” appearance [13]. According to a study, multinucleated giant cells with ground-glass nuclei and eosinophilic inclusions are often seen from a histological standpoint. At least 30% of instances of gastrointestinal bleeding are caused by HSV esophagitis. Primary infection accounts for 60% of cases, latent infection for 10%-20%, and superinfection for 10%-20%. According to the study, those with a robust immune system usually have minor symptoms from primary infections [3].

In contrast, those with a weak immune system may experience more severe symptoms from latent infections caused by a dormant virus but might reawaken when the host's defenses are compromised. Significant erosions or isolated ulcers (linear, longitudinal, and deep) in the lower section of the esophagus were detected by esophagogastroduodenoscopy. A confirmatory biopsy verified the presence of inclusion bodies in the nucleus or cytoplasm and the tissue damage [14]. Mucosal friability, ulcerations, and pseudomembranes are endoscopic findings of bacterial esophagitis caused by nonspecific inflammation. Without the presence of fungus, viruses, malignancies, or recent esophageal surgery. found histological evidence of bacterial invasion in the esophageal mucosa or deeper layers. Bacteremia is a potential consequence of bacterial esophagitis (Table 1) [15].

**Table 1:** Global and regional HIV data which can infer trends that may relate to HIV esophagitis

Country/Region	Prevalence (millions)	Incidence (annual new cases, millions)	Mortality (annual deaths, millions)	Key Trends and Observations	References
Global	36.8	1.94	0.95	Declining mortality and incidence but increasing prevalence.	[16]
Sub-Saharan Africa	Highest	High	High	Highest burden, but varies significantly across specific countries.	[17]
North America	1.1 (approx.)	Stable with some increases	Declining	Increases mainly in MSM populations.	[18]
Eastern Europe	Moderate	Rising	Declining	Rising new cases despite global declines in other regions.	[18]
Asia	Moderate	Variable	Variable	Regional variations with some countries experiencing increases.	[18]
Latin America	Moderate	Stable to rising	Declining	Stable but with pockets of increases in certain areas.	[18]

Etiology

Several variables may cause infectious esophagitis: Infectious agents mainly consist of fungi, including *C. albicans*. Some members of the *Candida *family, including *C. galbarta*, *C. tropicalis*, and *C. parapsilosis*, may cause esophagitis, but they are uncommon. To name a few more fungal species, there are *Histoplasma*, *Aspergillus*, *Cryptococcus*, and *Blastomyces* [19]. Herpes simplex virus, varicella-zoster virus, human papillomavirus, Epstein-Barr virus, cytomegalovirus, and poliovirus are all examples of viruses. It must be recognized that people infected with *M. avium*-*intracellulare*, human immunodeficiency virus, cytomegalovirus, or herpes simplex virus have no known cause. The following bacterial examples are included in the list: normal flora, *Streptococcus*, *M. avium-intracellulare*, *Norcadia*, *Mycobacterium tuberculosis*, and *Staphylococcus*. Some examples of parasitic agents are *Leishmania donovani*, *Pneumocystis*, *Cryptosporidium*, Chagas disease, and *Trypanosoma cruzi*.

Antibiotic and steroid usage, radiation treatment, chemotherapy, cancer, and immunodeficiency diseases like AIDS are all potential causes of infectious esophagitis. Candida esophagitis is more common in older adults, and it is also linked to esophageal stasis, intoxication, malnutrition, and older age. Patients without a history of esophageal or systemic diseases may sometimes have sporadic manifestations of Candida esophagitis [20]. Dysphagia, or trouble swallowing, and odynophagia, or painful swallowing, are symptoms that may appear suddenly in certain instances of infected esophagitis. Symptoms, including nausea, vomiting, heartburn, and soreness behind the breastbone, might all manifest at the same time. Abdominal discomfort, lack of appetite, reduced body weight, and coughing are symptoms that patients may experience intermittently. Candida infections often cause infectious esophagitis. Herpes simplex virus and cytomegalovirus infection are other important causes. Rare instances of infected esophagitis may manifest in people with signs of systemic sepsis, low neutrophil counts, AIDS, burns, trauma, etc. Severe esophagitis caused by actinomycosis may manifest as deep ulcers and fistulous tracts that spread to many tissues, including the epidermis, tracheobronchial tree, pleural space, and mediastinum. The sulfur granules on endoscopic biopsy specimens confirm the diagnosis of infected esophagitis. Recurrent episodes of low CD4 count are the primary determinant for infectious esophagitis in HIV-positive people.

Alternatively, it has been suggested that people may have fungal esophagitis during seroconversion [21]. A congenital immunodeficiency disorder called chronic mucocutaneous candidiasis is associated with Candida esophagitis. Esophageal cancer, Achalasia, and progressive systemic sclerosis are some of the conditions that might cause fungal esophagitis because they interfere with the natural mobility of the esophagus. The main feature of HPV-induced esophagitis is the creation of small vesicles, which, when they burst, cause the mucosa to develop distinct, superficial ulcers. The host helps the ulcers heal in those with strong immune systems. Localized ulceration may progress to widespread hemorrhagic esophagitis in patients with severe immunodeficiency. Necrotic herpetic ulcers may be severely infected with candidiasis. Mediastinal lymph nodes next to the esophagus may deteriorate and impact the esophagus, leading to tuberculous esophagitis. In addition, microbes in the mouth are the source of bacterial infections of the esophagus, which often affect people with compromised immune systems and include several kinds of bacteria. This syndrome is common in severely granulocytopenic individuals, although it frequently goes undiagnosed due to the difficulty of detecting bacteria in routine histologic tests. Bacterial infections often coexist with more obvious viral or fungal infections in these individuals. Bacterial and fungal esophagitis are more likely to occur in patients using proton pump inhibitors, which reduce the stomach's acid production. Endoscopic biopsy confirms the diagnosis when it finds bacterial clusters with necrotic epithelial cells in the specimens. Antibiotics, which may kill various germs, are part of the treatment plan. Less frequent causes of infectious esophagitis include *Staphylococcus*, *Klebsiella*, *Blastomyces*, *Cryptosporidium*, *Torulopsis glabrata*, *Streptococcus*, and *Lactobacillus acidophilus*; nevertheless, viruses and fungi are the most prevalent culprits [22].

Pathophysiology

**Figure 1:**
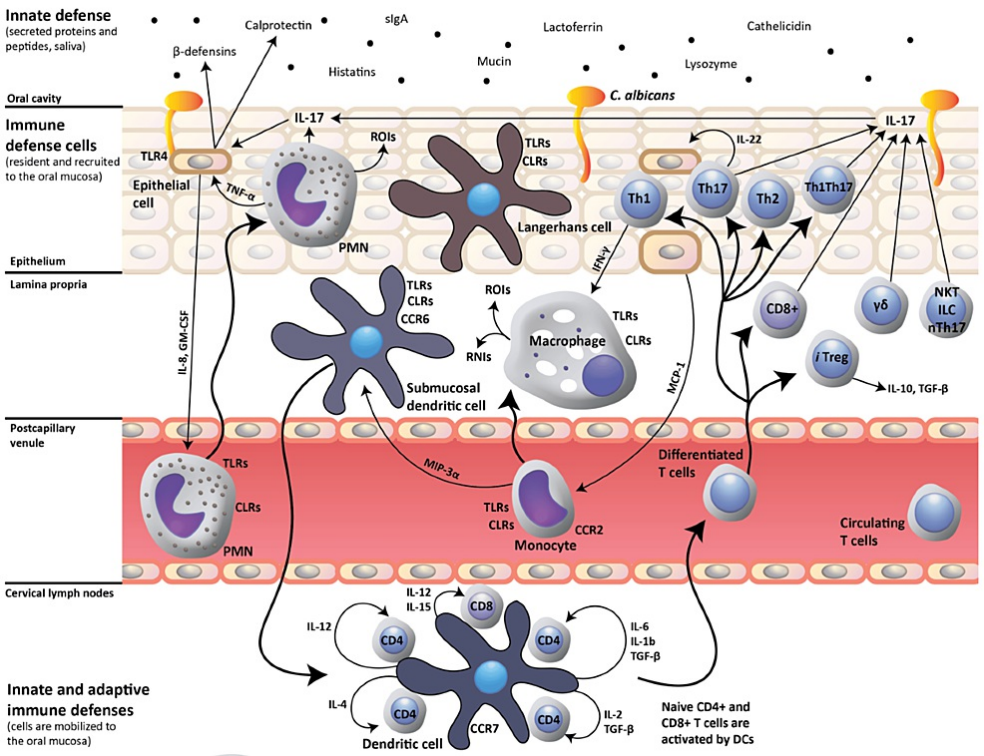
Host response to oral Candida albicans infection An effective host defense against oral *C. albicans* infection relies on the activation of T helper 17 (Th17) cell-mediated adaptive immunity by dendritic cells (DCs). This response stimulates the production of interleukin-17 (IL-17), which enhances the innate production of mucosal antimicrobial peptides (such as beta-defensins and calprotectin) by epithelial cells. IL-17 also increases the production of IL-8 and granulocyte-macrophage colony-stimulating factor (GM-CSF) in epithelial cells, leading to the mobilization of neutrophils to the oral mucosa. Additional innate-like cell groups, including gamma delta (γδ) T-cells, natural killer T (NKT) cells, innate lymphoid cells (ILCs), and nonclassical Th17 cells (nTh17 cells), contribute to this mucosal response by producing IL-17 as well. The interaction involves C-type lectin receptors (CLRs), reactive nitrogen intermediates (RNIs), reactive oxygen intermediates (ROIs), and toll-like receptors (TLRs). Reproduced under the terms and conditions of the Creative Commons Attribution license from Ref. [26]. Copyright © 2015 by the authors; licensee MDPI, Basel, Switzerland.

Various microorganisms, including fungi, bacteria, parasites, viruses, and others, may cause infectious esophagitis (Figure 1). Although those with weakened immune systems are more likely to experience this illness, it may also appear in otherwise healthy people of any age [23]. Infectious esophagitis is most often caused by fungi and viruses, but bacteria are the least likely culprit. While naturally occurring in the mouth, *C. albicans* may cause issues when numbers increase, as happens when patients take antibiotics or corticosteroid medicine, which weakens the immune system. The squamous epithelium is the main target of the herpes simplex virus, which leads to vesicles and, eventually, ulcerations in cases of viral esophagitis. Not only may coronavirus cause viral esophagitis, but varicella-zoster and Epstein-Barr virus can also cause it. Numerous anomalies in the immune system put individuals at risk for opportunistic infections, which may manifest as neutropenia, decreased chemotaxis and phagocytosis, impaired T-cell lymphocyte function, and alterations in humoral immunity. Due to impaired immune function, infectious esophagitis may be a severe problem for those with systemic illnesses, including alcoholism, diabetes, adrenal dysfunction, or late age. Using steroids, radiation, cytotoxic compounds, and immunological modulators can lower the host immune system's functioning. Commensal organisms may be more invasive due to the disturbance of normal bacterial ecology brought about by mucosal protective barriers and medications [24]. Candida esophagitis, an infectious disease of the esophagus, is primarily caused by the fungus *C. albicans* and predominantly afflicts individuals with compromised immune systems, including those with HIV/AIDS, cancer patients undergoing chemotherapy, or individuals receiving immunosuppressive drugs for organ transplants [19]. Cervical and herpes simplex virus esophagitis are two examples of viral esophagitis. Mycoses, such as actinomycosis and tuberculosis, may cause bacterial esophagitis. Other conditions that have the potential to cause Candida esophagitis [25] include the overgrowth of fungi in the esophagus, a lack of effective cell-mediated immunity, or both. Fungal overgrowth may arise due to esophageal stasis because abnormal function is a hallmark of esophageal motility disorders such as achalasia. Potential causes include scleroderma and mechanical issues like strictures [22].

Different types of esophagitis have different historical outcomes. Proton pump inhibitor-responsive eosinophilic esophagitis may primarily appear as esophageal food impaction [27]. A congenital immunodeficiency disorder called chronic mucocutaneous candidiasis is associated with Candida esophagitis. Fungal esophagitis may develop due to achalasia, esophageal cancer, or progressive systemic sclerosis, impairing the esophagus's normal motility. As a result of the creation of small blisters, which burst with time, distinct superficial ulcers may appear on the mucosa in cases of HPV-induced esophagitis [28]. The host helps the ulcers heal in those with strong immune systems. Localized ulceration may progress to widespread hemorrhagic esophagitis in patients with severe immunodeficiency. Extensive infection of necrotic herpetic ulcers may occur with candidiasis. Mediastinal lymph nodes next to the esophagus may deteriorate and impact the esophagus, leading to tuberculous esophagitis. Also, several different kinds of bacteria may coexist in the mouth and cause an esophageal infection, often affecting those with compromised immune systems. Because bacteria are difficult to detect with conventional histologic testing, the prevalence of this illness is frequently underreported in individuals with a significant reduction in granulocytes. Patients with these more easily identifiable diseases sometimes have bacterial, viral, or fungal infections. Bacterial and fungal esophagitis are more likely to occur in patients using proton pump inhibitors, which reduce the stomach's acid production. Endoscopic biopsy confirms the diagnosis when clusters of bacteria are seen in the specimens combined with necrotic epithelial cells. Antibiotics, which may kill various germs, are part of the treatment plan [29,30]. Viruses and fungi are the most common causes of infectious esophagitis. Yet, on rare occasions, infections can be caused by *Staphylococcus*, *Klebsiella*, *T. glabrata*, Blastomyces, Cryptosporidium, *Streptococcus*, or *L. acidophilus*.

Diagnosis strategies

It is essential to look at the diagnostic and diagnostic factors for each kind of infectious esophagitis when assessing this condition's diagnosis. This will give you a thorough grasp of all the essential things to consider when choosing suitable therapies for your patients.

Diagnostic Considerations

Always keep in mind that a systemic condition, such as AIDS, scleroderma, pemphigus, or systemic lupus erythematosus, might cause esophageal symptoms. In addition, the correct medicine should be administered after considering any cardiac causes that can cause chest discomfort. The patient should be hospitalized for additional evaluation if the diagnosis is unclear. Be cautious not to mistake pain in the chest from a heart attack for pain in the throat. There may be shared experiences of pain, particularly among women and the elderly [31]. Some of the following conditions have been shown to have symptoms similar to esophagitis.

Factors to Consider When Diagnosing Different Types of Infected Esophagitis

The following is an analysis of the diagnostic criteria for the various forms of infected esophagitis.

Diagnosis of Candida esophagitis: A few little cuts and scrapes. One symptom of superficially spreading cancer is a continuous patch of sickness that looks like nodular mucosa with fuzzy borders. Candidal infections may cause lesions resembling esophageal indissoluble effervescent particles and debris. Effervescent granules should not be used during a double-contrast examination in cases when infectious esophagitis is suspected [32]. HIV esophagitis should not be ignored as a potential problem for HIV-infected persons, especially the advanced cases of HIV disease. Candida esophagitis occurs rather frequently in patients with HIV being immunocompromised, mainly in those with CD4 T cells lower numbers. Concerning early anti-fungal therapy, the esophageal biopsy is helpful as a guide according to the directions by Vaezi et al. before discovery during endoscopic examination. CMV, or cytomegalovirus, is an essential cause of infectious esophagitis among HIV patients as well [33]. Fine endoscopic surveying followed by a fibroscopic biopsy is thus a necessary part of the diagnosis, highlighting the necessity of the CMV virus as a pathogen in immunocompromised individuals. The highest numbers of esophageal Candida infections times have been reported, reflecting a borderline relation between antiretroviral therapy adherence and growth in the population of both old and young patients with HIV. As expected, age, HIV status, and corticosteroid use have a strong connection to esophageal candidiasis [34]. Fresh diagnostic methods, for instance, molecular viral assays, make it possible for an early diagnosis of HIV and esophagitis. This will indirectly benefit others who HIV test positive and are at higher risk of the same infection [35]. Recent breakthroughs in the knowledge of how HIV relates to the esophagus point to the need to carry out comprehensive testing and evaluation, with endoscopies, biopsies, and management of the underlying HIV condition being included. Proper, timely diagnosis and treatment, taking into account the specific pathogens process that the patient is afflicted with are very important factors for patient outcomes in the same population group.

Diagnostic factors for HIV esophagitis: Endoscopy is necessary to exclude CMV esophagitis as a diagnosis before confirming HIV esophagitis since relying only on radiographic and clinical criteria is insufficient to distinguish between the two forms of ulcers. Supplementary materials, such as esophageal viral cultures, brushings, or biopsy specimens, may be required to validate the diagnosis and determine the appropriate therapy for the patient. Patients may acquire several tiny aphthoid lesions while experiencing the brief symptoms of chills, fever, malaise, and rash that are characteristic of early HIV infection. Later, one may see extensive and profound ulcers of several centimeters. Patients with large ulcers are at risk of experiencing bleeding, perforation, fistula development, and superinfection [36]. It is essential to distinguish between HIV esophagitis and other conditions because HIV esophagitis requires antiviral solid drugs such as ganciclovir for treatment. However, oral steroid therapies are generally beneficial for the majority of persons with HIV esophagitis. Therefore, it is advisable to do an endoscopic examination before starting the patient's treatment [1]. Most epithelial lesions and cancers on the mucosal surfaces of the skin are caused by the human papillomavirus, a non-enveloped circular DNA virus with two strands. People infected with human papillomavirus over an extended period, especially those who have several sexual partners, are very likely to get other subtypes of the virus, which number over a hundred. Different types of human papillomavirus infections include epidermodysplasia verruciformis and non-genital/cutaneous and mucosal or anogenital infections. Testing for viral deoxyribonucleic acid may be necessary to confirm the diagnosis of latent HPV lesions, even if the clinical lesions of HPV may be seen clinically in certain situations. Most cases of human papillomavirus infection are latent, and lesions caused by the virus often manifest clinically as warts rather than cancers [37]. Cancers of the larynx, mouth, lungs, and anogenital area are now thought to be caused by the Human Papillomavirus. Condylomata and low-grade precancerous lesions are common symptoms of HPV subtypes six and 11, which are considered low risk. Human papillomavirus types 16 and 18 are associated with an increased risk of developing cancer by causing high-grade intraepithelial lesions. Stressing the point, HPV does not cause cancer on its own. Cancer may start with a lack of folate. Still, it requires several other conditions to progress, including smoking, a compromised immune system, being pregnant, and exposure to UV radiation [38,39]. No outward signs or symptoms are seen in cases with HPV-induced esophagitis. The illness often manifests as lesions in the middle to lower part of the esophagus. Some possible manifestations of these lesions are red, inflamed areas, white patches, nodules, or many lesions that resemble fronds. Immunohistochemical stains may detect cellular alterations such as koilocytosis, big cells, and cytologic atypia, which diagnose HPV esophagitis when examined under a microscope [40].

Controlling and Treating Infected Esophagitis

The immunological status of the patient, the intensity of the disease, and the probability of complications dictate the course of treatment for infectious esophagitis [41]. Medical therapy aims to lessen the likelihood of unfavorable health consequences and treat the underlying cause. What follows is an analysis of the methods used to treat infectious esophagitis in its many manifestations [42].

Management of esophageal candidiasis

Anti-fungal medicine is often used when treating candidiasis that has spread to the oral cavity, esophagus, or throat. Here is a breakdown of the therapies: Oral amphotericin B, nystatin, and clotrimazole are some active components in topical treatments. Drugs like fluconazole and itraconazole are absorbed orally. Injections or intravenous drips provide medications, including flucytosine, amphotericin B, and fluconazole.

Oral administration of antifungal medications for seven to 14 days is standard for treating moderate to severe oral fungal infections. Common options include nystatin, miconazole, and clotrimazole, with fluconazole often used as the first line of defense. If fluconazole is ineffective, an alternative antifungal should be considered, especially for treating Candida esophagitis in patients who experience adverse effects or do not respond to fluconazole [43,44].

Treatment of HIV esophagitis

When treating HIV esophagitis, it is common practice to combine antiretroviral medication with oral corticosteroid treatment for a minimum of one month. When acyclovir fails to alleviate the symptoms of VZV esophagitis, other antiviral medications such as famciclovir or foscarnet may be used [45]. When treating EBV esophagitis, acyclovir is the drug of choice. Maintenance therapy may be required for the patient to successfully manage oral hairy leukoplakia [46]. Due to the lack of symptoms often experienced by HPV esophagitis, treatment is sometimes deemed unnecessary. The therapeutic efficacy of medications like bleomycin, etoposide, and systemic interferon alfa is variable [3]. When treating *M. tuberculosis* esophagitis, patients with a robust immune system often get standard antituberculous medication [47]. While immunocompromised patients often get infections caused by natural flora, healthy people seldom do. Staphylococcus aureus, Staphylococcus epidermidis, Streptococcus viridans, and Bacillus species are typical components of polymicrobial infections in patients with bacterial esophagitis. A broad-spectrum beta-lactam antibiotic is used with an aminoglycoside to treat bacterial esophagitis. Recognizing that the treatment is adjusted based on response and cultural findings is essential [3,48].

No statistical difference in the effectiveness of fluconazole concerning other first-line treatments employed in the management of Candida esophagitis in HIV patients was demonstrated. Furthermore, he is conspicuous about managing infections related to *C. albicans*; nevertheless, the resistance among the non-albicans *Candida* type seems to be an issue. The utility of therapeutic strategies is further emphasized by the fact that susceptibility testing should be routine, along with determining alternative anti-fungal treatments based on respective resistance profiles [33]. Although the literature review did not unearth new drugs stemming from 2015 onward for measuring CMV in HIV esophagitis, the existing regime of ganciclovir followed by foscarnet for the instances of resistance continues to be the approach recommended for the treatment of this condition. Analyzing the resistance and serious side effects is critical to creating successful outcomes in the treatment [49]. Stuehler et al. made significant contributions to unraveling human immunodeficiency virus-infected people's interconnected immune system recovery patterns after esophageal Candida infection. The study stressed the need for cART to start as early as possible, and its continuity is a must to restrict immune defects and mucosal recovery. These findings undercoated the complex pathophysiology of the opportunistic infection [50]; other than these diseases, aPDT (AMPT) was shown as a new therapeutic way for treating HIV-related oropharyngeal candidiasis. Zeng et al. recently synthesized a meta-network analysis putting forward the crucial role of econazole (CND) in improving the cure rates and reducing relapse ratio compared to old-fashioned drugs such as fluconazole. However, its efficacy against esophageal candidiasis needs more observation [51]. This article evidences the power of well-known and new efforts for the treatment of HIV esophagitis. The relevance of immediate diagnosis and effective treatment regimen patterns because of the resistance profile and new techniques like photodynamic therapy (PDT) constitute a holistic tract toward high-quality patient management.

Infectious esophagitis epidemiology

Although adults often have esophagitis, the most common kind is reflux esophagitis, which is uncommon in children. The majority of cases of infectious esophagitis are caused by *Candida*. Approximately 44% of the population has reflux esophagitis at least once a month, with 10% reporting everyday symptoms [52]. Prevalence in association with other disorders. Although fewer than 5% of the population has symptomatic infectious esophagitis, the prevalence is much greater in those with AIDS, leukemia, and lymphoma. Infectious esophagitis caused by herpes simplex virus is the second most common kind. It has been seen in around 1% of patients with impaired immune systems and as high as 43% of patients in trials conducted after death [53].

CMV may also cause esophagitis. Worldwide, asymptomatic CMV infection is common, and a large percentage of the population has been exposed to the virus [54]. People with a competent immune system are not afflicted by CMV, in contrast to HSV esophagitis. People with AIDS make up the vast majority of cases of CMV esophagitis. The incidence of CMV esophagitis and other types of infectious esophagitis has declined in AIDS patients since the advent of HAART.

In contrast, solid organ transplant recipients have been shown to have an increased risk of CMV esophagitis. As early CMV prophylaxis becomes more widely used, the onset of sickness is postponed, contributing to this upsurge. When no other viral reasons can be discovered, AIDS patients may develop large ulcers in the esophagus, which are called idiopathic HIV ulcers. The presence of HIV-like viral particles in the ulcer lesions, as verified by electron microscopy, has led researchers to assume that HIV causes these ulcers. With CD4 levels below 100 cells/µL, chronic AIDS has been identified in most individuals. Some people who have HIV ulcers may have recently had seroconversion; however, it does happen. Although esophageal ulcers constitute about 40% of all ulcers reported in the AIDS community, they are often underrecognized (Table 2) [55].

**Table 2:** Overview of recent research on treatments for HIV esophagitis

Year	Intervention Type	Key Outcome	Study Details	Cure/Improvement Rates	Acceptability/Relapse Info	Source
2021	aPDT with laser irradiation and methylene blue	Highest cure rate for oropharyngeal candidiasis	Network meta-analysis including multiple RCTs comparing various anti-fungal treatments	Significantly higher than fluconazole	Lower relapse rates compared to fluconazole	[51]
2022	Updated antiretroviral therapy guidelines	Emphasizes integrase strand transfer inhibitor-containing regimens as mainstay	Recommendations based on critical evaluation of new data covering a broad range of HIV-related treatments	NA	NA	[56]
2020	Acyclovir treatment for herpetic esophagitis	Effective in improving symptoms in immunocompetent hosts with herpetic esophagitis	Case series reporting significant symptom improvement after acyclovir treatment	Complete resolution noted	Good tolerability with acyclovir	[57]
2021	Valacyclovir for HSV-induced esophageal ulcers	Complete resolution of esophageal ulcers	Case report on an immunocompetent middle-aged woman with acute gastrointestinal bleeding secondary to HSV-1	Complete resolution after 10 days of treatment	Effective and well-tolerated	[58]
2020	HPV-associated esophageal ulcer management	Effective management with antiretroviral agents and proton pump inhibitors	Single case study on an HIV-infected patient with HPV-associated esophageal ulcer	Significant symptom improvement	HPV detection and management outlined	[59]

## Conclusions

To summarize, it is common for individuals with advanced HIV/AIDS to develop esophagitis, which is frequently caused by opportunistic infections such as those related to *C. albicans*. This condition is characterized by inflammation, the formation of esophageal ulcers, and other severe symptoms that can lead to significant morbidity and mortality due to compromised immune defenses. Effective management of this condition requires timely diagnosis, the administration of antifungal medications, such as fluconazole and amphotericin B, and the management of lifestyle factors like alcohol and tobacco consumption. Proactive and continuous therapy is essential to mitigate the risks associated with this severe condition.
